# What validated instruments, that measure implementation outcomes, are suitable for use in the Paediatric Intensive Care Unit (PICU) setting? A systematic review of systematic reviews

**DOI:** 10.1186/s13012-024-01378-4

**Published:** 2024-10-10

**Authors:** Elizabeth Dodds, Sarah Redsell, Stephen Timmons, Joseph C. Manning

**Affiliations:** 1 Paediatric Critical Care Unit, Nottingham University Hospital NHS Trust, Nottingham, UK; 2 School of Health Sciences, University of Nottingham, Nottingham, UK; 3 School of Healthcare, College of Life Sciences, University of Leicester, Leicester, UK; 4 Centre for Children and Young People’s Health Research (CYPHR), University of Nottingham, Nottingham, UK; 5 Health Service Management, Nottingham University Business School, Nottingham, UK; 6 Nottingham Children’s Hospital, Nottingham University Hospitals NHS Trust, Nottingham, UK

**Keywords:** Outcomes, Instrument validation, Evidence-based practice, Healthcare

## Abstract

**Background/aims:**

The measurement of implementation outcomes can establish the success of implementing evidence into practice. However, implementation outcomes are seldom measured in acute healthcare settings, such as Paediatric Intensive Care Units (PICU), and if they are used, are likely to be non-validated, site or intervention-specific measures. To address this literature gap, this systematic review of systematic reviews aims to identify validated instruments to measure implementation outcomes of new EBP interventions in a PICU setting.

**Methods:**

A systematic review of systematic reviews was conducted in two phases. *Phase One:* Five electronic databases were searched between 06/10/22 and 14/10/22. Systematic reviews were selected using pre-determined eligibility criteria. Methodological quality was assessed using the Critical Appraisal Skills Programme tool and a data extraction table was used to allow further synthesis. *Phase Two:* Secondary eligibility criteria were used to extract and review instruments from the systematic reviews selected in Phase One. Instruments were analysed and mapped to the Consolidated Framework of Implementation Research (CFIR).

**Results:**

*Phase One:* Searches resulted in 3195 unique papers. Five systematic reviews were eligible for inclusion. All examined the psychometric properties of each instrument, utilising different methods to do so; three considered their pragmatic or usability properties; and one identified instruments that were transferrable to different settings. Each systematic review identified that most included instruments had limited evidence of their validity or reliability and had poor psychometric properties. *Phase two:* 93 instruments were screened, and nine were eligible for analysis. After analysis and CFIR mapping, two instruments were identified as potentially adaptable to the PICU setting.

**Conclusions:**

The methodological quality of implementation outcome measurement instruments is inadequate, warranting further validation research. Two instruments were identified that cover multiple CFIR domains and have scope to be adapted for use when implementing evidence-based practice into the PICU. Further work is needed to adapt and further validate an instrument for use in practice.

**Trial registration:**

For transparency of procedures and methods, the protocol for this systematic review was registered with PROSPERO (registration number CRD42022361638L).

Contributions to literature

- A systematic review of systematic reviews (SR of SRs) provides a new contribution to the existing literature, utilising a rigorous approach to examine existing research on implementation outcome measurement tools providing an further appraisal of the identified findings.
- Whilst the findings of the SR of SRs identify that existing implementation outcome measurements are of predominately poor methodological and psychometric quality; it was also identified that providing further validation and development to an existing instrument would be of greater benefit than developing a new instrument.
- The rigorous and replicable methods used enabled the identification of two instruments with scope for use in an acute healthcare setting such as the PICU. Furthermore, the process supports the adaption by other researchers for use in their settings.

## Background

Whilst there is considerable investment in improving the quality of care in healthcare, it is recognised that if implementation processes are flawed then evidence-based practice (EBP) and innovations are less likely to meet their potential [1]. As such, measuring implementation outcomes can identify success in implementing new EBP into practice [2]. The field of implementation outcome research has expanded considerably since the seminal work undertaken by Proctor et al. in 2011 [3]. There is improved recognition of the concept and the framework created has been used by multiple researchers seeking to create methods of measuring implementation outcomes. However, a scoping review considering a decade of implementation outcome research [2] highlighted that fewer than a third of the identified papers from this review were empirical and those that were, looked at a single EBP intervention in a specific setting. This is further iterated by a paper identifying that work on implementation outcome measures is likely to be crude and lacking in rigour [4]. As such, research is struggling to capture the realities of implementation science in practice, i.e. how to implement and sustain EBP in fast paced and unpredictable healthcare environments [2].

Considering the need to address the lack of implementation outcome measures in all healthcare settings, the Paediatric Critical Care Unit (PICU) setting provides a useful case study with wider applicability. Rising morbidity rates in intensive care units (ICUs) can be attributed to various factors, potentially including the challenges associated with implementing evidence-based practice (EBP) in this demanding clinical environment [5]. PICUs encounter further hurdles due to the diversity of their patient population, increased error risks, and complex ethical and financial considerations associated with conducting trials in this specialized context [6, 7].

Within healthcare settings, there is a recognised need to understand why interventions with positive clinical outcomes may fail while ineffective ones persist [8, 9]. Considering the PICU, an effort was made to address these knowledge gaps by utilizing an implementation framework to guide the integration of EBP into PICUs, [6]. However, this research did not extend to measuring the outcomes of the implementation strategy.

PICU implementation outcomes, in common with most implementation outcomes, are seldom measured, and when they are, they often rely on non-validated methods and potentially flawed designs [10]. Alternatively, smaller-scale qualitative methods are employed [11], providing valuable insights but being less feasible for larger interventions or settings lacking trained qualitative researchers.

To address this gap in the literature, this systematic review (SR) of SRs aims to identify validated instruments to measure implementation outcomes of new evidence-based practice interventions in acute healthcare settings, with applicability for use in the PICU. In recognition of the novel nature of this work and the broader literature gaps within implementation research, this paper aims that the methods used could be adapted for use by researchers from different healthcare settings.

## Methods

### Design

After identifying two published systematic reviews (SRs) that evaluated implementation outcome measurement instruments used in healthcare settings [12, 13], the decision was made to undertake a SR of SRs to enable a single synthesis of all available and relevant evidence. This is a Cochrane Collaboration recognised method [14] and this review contains the five components necessary for a Cochrane Overview. There is a formulated research question and only systematic reviews are included. Explicit and reproducible methods were used to identify the systematic reviews [14]. However, whilst this methodology was followed where possible, there are key differences between this review and those detailed in the Cochrane Overview meaning that not all aspects of the methodology were relevant. The nature of Cochrane reviews is that they review empirical research looking at healthcare treatments or interventions, usually RCTs, whereas the primary purpose of this review is to evaluate the development and use of measurement instruments, so the methodology is necessarily adapted in some areas to reflect this and this will be documented. Other studies examining the methodology of systematic review of reviews will be taken into consideration to ensure this review is methodologically sound [15–17].

For transparency of procedures and methods, the protocol for this systematic review was registered with PROSPERO (registration number CRD42022361638L). Reporting was based on the PRIOR checklist [18].

### Data source

Comprehensive electronic searches of MEDLINE, EMBASE, CINAHL, PsycINFO and PubMed were conducted between 06/10/22 and 14/10/22. The full search strategy has been published on PROSPERO. Deviations from the published protocol included not searching the Web of Science and The Cochrane database following advice from the information scientist as Web of Science expected to yield many duplicates with PubMed and Cochrane to not to yield any relevant results due to study design. Reference lists of included reviews were also searched for any additional eligible papers.

### Inclusion criteria

The inclusion/exclusion criteria for study selection were based on discussion with the review panel and are listed below in Table 1. In recognition of one of the key limitations of systematic reviews of reviews, that there can be a significant time lag between primary research, the initial systematic review and then the systematic review of reviews [16], the publication date was limited to 2012 as this post-dates the seminal work on the taxonomy of outcomes [3] therefore the search date was 01/01/2012 to present day.

**Table 1:** Eligibility criteria

	**Inclusion**	**Exclusion**
**Year of Publication**	2012 to present day. No date limit for studies with systematic reviews	Pre 2012
**Study type**	Systematic review Meta-analysis Cochrane review (eligible protocols can be screened for full text to establish whether the review has been completed/published)	Realist review Scoping review Studies using any other review method Empirical research Editorial pieces
**Language**	English language (or translated version available) review Instrument outcome measures available in and developed for the English language	No translation available Instrument outcome measures designed specifically for other languages
**Outcome measures**	Reviews reporting instruments measuring implementation outcome measures e.g. based on Proctors taxonomy of outcomes; Consolidated Framework of Implementation Research; RE-AIM; Normalisation process theory	Reviews reporting clinical effectiveness and/or patient reported outcomes
**Participants**	Reviews reporting instruments measuring implementation outcomes of an intervention for/with healthcare professionals	Reviews reporting instruments that only use patients or public as participants
**Setting**	Any healthcare setting	
Methodological Strength	Reviews that consider the methodological quality/psychometric strength of the instruments	

### Selection of studies and data extraction

Search results from electronic databases were downloaded to EndNote referencing software and duplicates removed. The lead reviewer (ED) screened the titles and abstracts to identify studies that fit the eligibility criteria. A selection of 25% of these were screened independently by a second member of the review panel (KW). All full texts were then screened and read by ED and KW independently with any discrepancies discussed with the full review panel and a consensus reached. The eligible papers had their references screened and where any published protocols for a systematic review were found, a search was undertaken to establish whether the full review had been published.

Data were extracted into a summary of findings table with categories including: author; title; year of publication; healthcare setting; number of instruments found; implementation framework used; psychometric and pragmatic measures used.

To assess the methodological quality of each eligible review, different tools were considered. The CASP (Critical Appraisal Skills Programme) tool was selected as it provides a succinct but effective method of appraising systematic reviews, focusing on the rigour and validity of the results without specifying the use of a specific type of primary evidence [19].

### Data synthesis

There were two phases to the data synthesis that link to the two objectives formed from the review question.

#### Objective one

To summarise systematic reviews that assess the methodological quality of instruments used to measure implementation outcomes in the healthcare setting.

#### Phase one

A narrative summary of the eligible studies was undertaken with emphasis on their search strategy and methodological quality, guided by the CASP tool analysis undertaken on each study. At this stage, the studies were looked at as a whole rather than considering the individual instruments examined within each study. The findings from the studies were organised, any patterns and relationships found were analysed and the factors that may explain similarities and differences in the included studies were considered. Specific analysis was given to how each paper was assessed and how the methodological and psychometric quality of the identified instruments were rated.

#### Objective two

To identify one, or more, reliable and validated implementation outcome measurement instruments that is applicable for use to measure implementation outcomes in the PICU setting.

#### Phase two

A second set of eligibility criteria (see Table 3) was used to extract and review relevant implementation outcome measurement instruments from the systematic reviews. Two reviewers undertook this independently to ensure rigour, with any discrepancies taken to a third reviewer to be resolved. Instruments that fit the criteria were mapped to the Consolidated Framework for Implementation Research (CFIR) and further analysed.

The use of the CFIR was chosen as it is a meta-theoretical framework, providing constructs to identify the influences on implementation, organise findings and analysing important processes and outcomes [20]. Mapping the eligible instruments to the domains and constructs enables a clear visual guide of which aspects of implementation are covered by each instrument and will thus help inform the decision on whether one instrument that covers a broad range of constructs would be more beneficial, or two or more separate instruments that each cover specific constructs in more detail may be appropriate.

## Results

### Phase one

#### Identification of relevant reviews

**Fig. 1:**
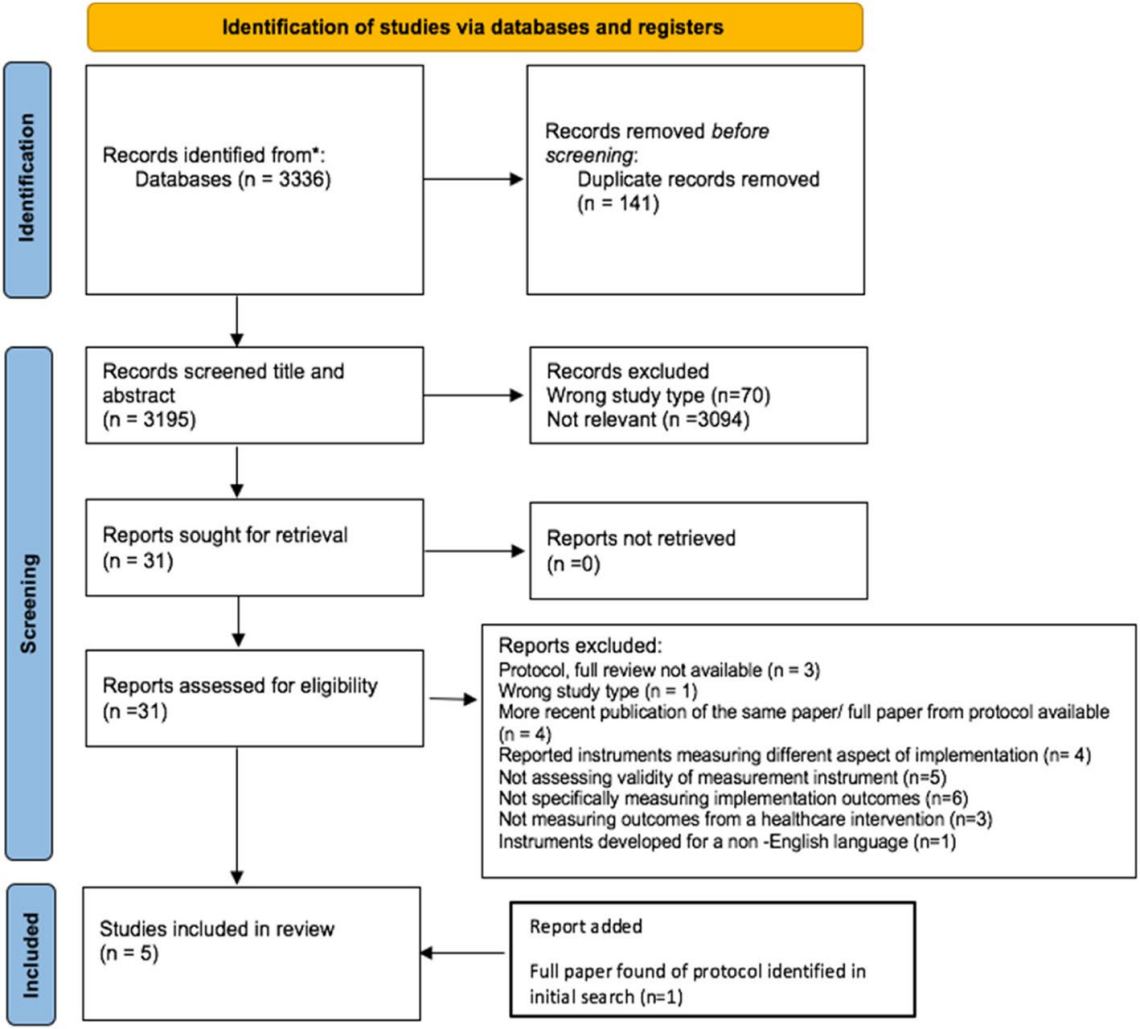
PRISMA flow diagram for database search

A total of 3195 unique citations were identified. After the title and abstract were screened, 3164 were excluded, leaving 31 that were retrieved for full-text screening. Four of these were deemed eligible for inclusion and a further one was added after a protocol was identified in the 31 papers for which the completed review had been published but had not been identified in the initial searches. Three reviews were discussed with the review panel. Two were included [21, 22] after a discussion regarding the relevance of including reviews considering instruments that measure the implementation outcomes of healthcare policies, as opposed to interventions. One review was excluded as it only focused on the outcome of fidelity and the decision was made that the reviews should consider an implementation outcome framework (for example, Proctor’s taxonomy (2011)) as a whole, rather than a limited Sect. [23] as the frameworks provide a more thorough and rounded view of implementation, rather than a more narrow focus on one aspect. Figure 1 shows the PRISMA flow diagram for the selection process.

#### Characteristics of included reviews

See Table 2 for a summary of findings. Of the five included SRs, three were conducted in the USA [13, 21, 22], one in the UK [12] and one in Australia [24]. Regarding the identified instruments, all stated that the highest percentage of instruments were developed in the USA, with the others from high-income countries such as Canada, Australia and throughout Europe. Each review provided a breakdown of the setting of the included instruments (for example, outpatient, school or pharmacy based) apart from Khadjesari et al. [12].

**Table 2:** Summary of findings from included papers

Author and year	No. of papers	Measurement tools	Healthcare setting	Implementation framework	Search strategy	Location of studies	Psychometric measure	Usability measure
Khadjesari et al. (2020) [12]	58	55 Acceptability *n* = 33 Appropriateness *n* = 7 Adoption *n* = 4 Feasibility *n* = 4 Penetration *n* = 4 Sustainability *n* = 3	Physical health (not specified whether primary, tertiary etc.)	Proctor’s taxonomy of outcomes	3 sets of search terms 1) Implementation literature 2) implementation outcomes from Proctor 3) measurement properties of the instruments	Predominately high-income countries, majority from USA (*N* = 22)	COSMIN and ConPsy	Usability Scale
Mettert et al. (2020) [13]	83	102 Acceptability *n* = 32 Adoption *n* = 24 Appropriateness *n* = 6 Feasibility *n* = 18 Fidelity *n* = 18 Penetration *n* = 9 Sustainability *n* = 13	Mental and behaviour health, predominately in the outpatient community setting and half covered general mental health or substance abuse.-	Proctor’s taxonomy of outcomes	4 core levels for search terms. 1) terms for implementation. 2) terms for measurement. 3) terms for evidence-based practice. 4) terms for behavioural health. A 5th layer was added for the Proctor et al. taxonomy of outcomes	86% were from the USA (*n* = 85), with the remaining predominately high-income countries	PAPERS (Psychometric and Pragmatic Evidence Rating Scale)	This was not measured. PAPERS does have usability measures, but these were not used in this study
Clinton-McHarg et al. (2016) [24]	65	51 These were mapped to the CFIR constructs, most frequently measured were: relative advantage, networks and communication, culture and implementation climate	Public Health, including education facilities, nursing homes, pharmacies, workplaces and broader community settings	Consolidated Framework of implementation research (CFIR)	The core search terms comprised of 1) terms for measurement. 2) terms for psychometric properties 3) the levels at which the measurement could occur, 4) the goals of research implementation, 5) the CFIR constructs	Predominately from the USA, (*n* = 28), with Canada and Australia also named. No further breakdown of countries	Criteria based on the Standards of Educational and Psychological Testing	Not measured
McLoughlin et al. (2021) [22]	67	86 *n* = 23 were classed as ‘large-scale tools’ (widely used and/or based on national samples), *n* = 63 were ‘unique tools’ – less frequently reported measures with smaller sample sizes. Top 5 constructs measured: Fidelity (*n* = 70) Actor relationships *n* = 45 Non-training resources *n* = 43 Leadership for implementation *n* = 42 Communication of policy *n* = 41 Many of the tools measured more than one outcome/determinant	School Health Policy. Most common topic was general wellness policy, followed by nutrition and physical activity	Proctor’s taxonomy CFIR Policy Implementation Determinants Framework	Terms based on 4 domains. 1) health, 2) public policy, 3) implementation, 4) measurement. Development of the search strings was based on implementation frameworks and policy research	69% were from the USA (*N* = 69) with a further 24.3% from Canada, Australia or Europe. The remaining countries were high or middle income	PAPERS	PAPERS, including brevity, cost, training, interpretation and readability
Allen et al. (2020) [21]	66	70 Top 5 constructs measured: Readiness for implementation 61% Organisational culture 23% Fidelity 26% Acceptability 24% Feasibility 17% Many of the tools measured more than one outcome/determinant. Of the 70, *n* = 38 were deemed mostly or partially transferrable to any health issue. Only the 38 were fully analysed	Health policy. The most common topics are mental health, general healthcare, schools and tobacco	Proctor’s taxonomy CFIR Policy Implementation Determinants Framework	Terms based on 4 domains. 1) health, 2) public policy, 3) implementation, 4) measurement Development of the search strings was based on policy implementation framework reviews and definitions of the included implementation outcomes	Full breakdown was not available for the 70. Of the 38 looked at in detail, *n* = 30 were from USA or Europe with the remaining tools used across the world, income status not listed	PAPERS	PAPERS, including brevity, cost, training, interpretation and readability

Four of the SRs used specific implementation outcome frameworks to search for and categorise the instruments [12, 13, 21, 22]. Two SRs solely used Proctor’s Taxonomy of Outcomes (2011) [12, 13]. The authors stated that the taxonomy was applied to ensure that all instruments fit the inclusion criteria of assessing an implementation outcome. The second two SRs [21, 22] also use the taxonomy for the same purpose but also use the CFIR and the Policy Implementation Determinants Framework [25]. The final SR [24] solely uses the constructs from the CFIR [20], which whilst also widely used within implementation research, does not focus on specific outcomes. A CFIR outcomes addendum is in the process of being developed [26] but the SR predates this.

#### Methodological strength

All five reviews used similar search strategies and had clear and justifiable eligibility criteria (Table 5). Two SRs published the protocols for the reviews before completion, providing further transparency of the process [27, 28]. A further two SRs were both undertaken by members of the same research team and stated that they based their review procedures on the method listed in the protocol of the Mettert et al. [13] review [28]. The Mettert et al. SR [13] yielded significantly more eligible instruments than the other reviews, 150, and 102 of which were eligible for psychometric rating. No explanation is provided for this but the combination of behavioural and mental health settings may have provided a broader search strategy thus yielding more results.

In the five included reviews, three different methods were used to assess the quality of the included papers. Mettert et al. [13] used a self-developed Psychometric and Pragmatic Evidence Rating Scales (PAPERS) [29], developed in response to a perceived gap in the literature in that no measures existed that were able to support the evaluation of the more specific and nuanced properties that fit the complexities of implementation science evaluations. Two further SRs identified also use this scale [21, 22]. Khadjesari et al. [12] also identified the same gap in the literature regarding psychometric measures. Firstly, they used the COSMIN checklist to assess methodological quality, specifically validity, reliability and responsiveness [30]. Secondly, they developed a contemporary psychometrics checklist (ConPsy) to assess psychometric strength more accurately. This aims to complement the COSMIN checklist and its psychometric strength is currently being evaluated as part of a separate study. Clinton-McHarg et al. [24] used the guidelines from the Standards for Educational and Psychological Testing [31] to assess methodological quality. PAPERS, COSMIN and ConPsy all provided a score for each instrument, the guidelines from Clinton-Mcharg et al. [24] provided a pass/fail mark.

#### Psychometric scores

Each review reported that the majority of instruments analysed had poor or inadequate psychometric scores. Khadjesari et al. [12]reported that of the scales reported reliability, only 8/62 scored a rating of excellent or good on the COSMIN checklist and only 11/63 which reported validity scored excellent or good. The ConPsy results were also low, out of a maximum score of 22, only 12 studies scored over seven, with the highest score recorded as nine. The Mettert et al. [13] review reported that out of 102 eligible instruments, 33 had no recorded psychometric properties and a further 39/102 scored between -1 to 2 out of a maximum of 36 on PAPERS. The highest recorded score was 12 and norms and internal consistency were the properties most likely to be measured. In the McLoughlin et al. [22] review, the instruments were split into two groups, those designed for large-scale purposes (e.g. regionally or nationally) and those designed as a unique tool for a specific policy. They found the large-scale tools showed marginally better psychometric scores due to high internal consistency and validity, however, the median PAPERS score across all measures was 0/36 due to the majority not providing psychometric information. In the Allen et al. [21] review, only 38/66 instruments that were assessed as being transferable to other settings were scored. The median PAPERS score was 5/36, with norms and internal consistency the most likely to be measured. Finally, in the Clinton-Mcharg et al. SR [24], of the 51 instruments, most demonstrated face/content and construct validity and internal consistency but only 3/51 achieved for test-rest reliability and 8/51 achieved for responsiveness.

#### Pragmatic strength

Whilst the PAPERS score was created by the team who undertook the Mettert et al. [13], they state in the protocol that they would not be applying the pragmatic rating scale as it was in the process of development. However, two other SRs use it [21, 22]. The scale uses the same scoring system as the psychometric scale and covers brevity, language simplicity, cost to use, training ease and analysis ease with a maximum score available of 20. Khadjesari et al. [12] also referenced PAPERS for their pragmatic scoring, however, they only score for brevity (referred to as usability) scoring it from minimal (over 100 items in the instrument) to excellent (under ten items). This is the only review to undertake further statistical analysis, using Spearman’s correlation to examine the relationship between the COSMIN and ConPsy scores and usability. Clinton-McHarg et al. [24] use the guidelines listed above to measure acceptability, feasibility and potential for cross cultural adaption, using a pass or fail score.

#### Pragmatic scoring

The Khadjesari et al. review looked solely at usability, i.e. number of items in an instrument. The number of items in each instrument ranged from 4- 68. 6/65 contained fewer than 10 (scored as excellent) and 55/65 contained between 10 and 49 items (scored as good). No correlation was found between usability and either COSMIN scale, and a small negative correlation was found between usability and ConPsy scores. Mettert et al. [13] did not use the pragmatic section of PAPERS but did report a number of items, ranking them in categories of 1–5 items (10/102) 6–10 items (10/102) or 11 or more items (82/102). No further analysis is provided. McLoughlin et al. [22] used PAPERS and found a median score of 10/20, the large-scale instruments and the unique instruments scored the same overall but had different areas of benefit, the large-scale ones were more likely to have training provided but were also much longer with an average of 150 items per instrument as opposed to an average of 73 items for the unique instruments. Allen et al. [21] also used PAPERS, again, analysing only the 38 instruments identified as transferrable to other settings. These had a median score of 11/20, averaging 4/4 for cost and 3/4 for brevity and language. Clinton-McHarg et al. [24] did not analyse the length of the instrument, instead assessed whether any papers reported the acceptability or feasibility of the instrument, 17/51 reported elements of these, with 5/51 reporting on length of time taken to complete the measure and 6/51 reporting the proportion of missing items. No other pragmatic data was reported.

### Phase two

#### Eligibility criteria for selecting instruments

Each review was assessed to establish that the instruments that they had reviewed were eligible for potential adaption to an acute healthcare setting such as the PICU. Three reviews were excluded [13, 22, 24] from phase two. All three provided a clear breakdown of the settings in that the instruments had been used, highlighting that they were predominantly outpatient, community, workplace or school-based, and for specific interventions, for example, smoking cessation or physical activity. This suggests that adaptability to an acute healthcare setting would be limited. Furthermore, all three reported the poorest psychometric results, and two lacked any pragmatic scoring [13, 24].

Instruments from the Allen et al. SR [21] were deemed acceptable for potential inclusion as the authors had undertaken analysis on each instrument to ascertain whether they could be transferable to different settings or contexts. Due to this, the 38 instruments that were assessed as fully or partially transferable were considered as they had the potential to be adapted to an acute healthcare setting. The Khadjesari et al. SR [12] provides some of the most detailed methodological and psychometric testing due to its use of both COSMIN and ConPsy scoring. Furthermore, the review specifically focused on instruments used in the physical healthcare setting and as this is inclusive of the PICU setting, suggests that instruments in this review were more likely to be appropriate for adaption. However, key contextual information is lacking in this review regarding the type of healthcare setting and as it is stated that most of the instruments were formed for a specific intervention [12], it is difficult to identify which may be adaptable for use with the PICU environment. As such, instruments in this review were considered for inclusion alongside those in the Allen et al. (2020) review, however, it was necessary to develop further inclusion/exclusion criteria (shown in Table 3) to narrow down the options and identify suitable options. The aim was to find an instrument that has been built for use, is adaptable to use in an acute clinical area, and has minimal or no cost involved with its use, as well as best scores on the COSMIN, ConPsy and usability and PAPERS scales. In recognition that both reviews highlighted that few instruments scored highly throughout, it was agreed by the co-authors that compromises could be made if an instrument fits the majority of the criteria but has one low score.

**Table 3:** Eligibility criteria for implementation outcome measurement instruments

	**Inclusion**	**Exclusion**
COSMIN	Score ideally good or excellent, fair accepted if fits other criteria	Reliability and validity scores of poor, or not assessed
ConPsy	Ideally 7 or above	5 or less
PAPERS psychometric score	A score of 5 or higher with more than one category measured	Score lower than 4 or only one category measured
PAPERS pragmatic score	10 or higher (minimum of adequate on each category)	9 or lower
Usability	Excellent or good	Fair or Poor
Setting	OECD countries Acute clinical setting, ward or ICU based	Primary health setting
Topic	Measuring the impact of an intervention or change in practice	Pre-implementation measures, e.g. readiness for change
Cost	Minimal or no cost for use	Significant cost per use
Respondents	Staff	Patients
Other	Patient and public involvement (PPI) in its development	

As both the ConPsy and PAPERS are newly developed scales, and due to the novel nature of this review, there is no established guide to determine what scores should be considered acceptable. Thus, the cut-offs were decided by considering the median scores in each review and giving in-depth consideration to scoring guidance for each scale. Any deviation from these criteria was discussed on a case-by-case basis. These decisions were all agreed upon by the full review panel.

#### Implementation outcome instrument selection

**Fig. 2:**
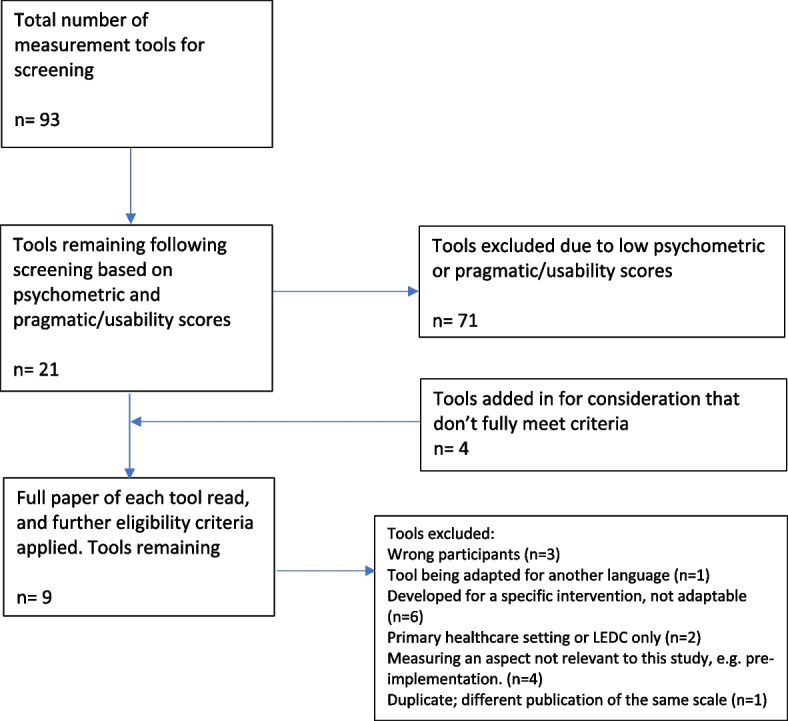
PRISMA Flow diagram for implementation outcome instrument selection

Initially, the instruments from the Allen et al. [21] and the Khadjesari et al. [12] SRs were screened by the psychometric and pragmatic eligibility criteria alone, yielding 21 results out of a potential 93. The title, scores and document characteristics available for each instrument in the reviews were then read in more detail and as a result, a further four were added for consideration. Three of these were from the same study [32] to be used together to cover three domains of the taxonomy so were considered for this purpose despite low COSMIN scores. A fourth was added as it scored highly for validity and usability in the Khadjesari et al. SR but had not assessed reliability [33], however, an older iteration of the same instrument had been identified in the Allen et al. SR [34] which met the PAPERS eligibility criteria. Both scores are included in the instrument characteristics table for reference (Table 4) however, only the most recent iteration was fully analysed, and the older paper was excluded as a duplicate. Finally, the full text of the paper associated with each instrument was read and the remaining eligibility criteria were applied. The process is detailed in Fig. 2 with explanations of exclusions. This left nine instruments for further analysis. However, as the three instruments by Weiner et al. [35] were designed to be used together, they will be considered as one instrument henceforth, so seven instruments will be discussed rather than nine.

**Table 4:** Summary of the implementation outcome measurement instruments

Title and Author	Taxonomy domain	COSMIN Reliability	COSMIN validity	ConPsy (CP)/ PAPERS Psychometric score	Usability (U)/ PAPERS Pragmatic score	Other Eligibility criteria	Adaptability for use
Person- Centred Healthcare for Older Adults (PCHOA) [36]	Acceptability	Excellent	Excellent	CP 7	U- Good [31]	Developed using PPI. Aimed at acute healthcare staff, focus on staff point of view (POV). OECD developed. No evidence of cost for use	On reading the scale it is focused towards caring for the older person, however over half of the questions could link easily to any healthcare setting including PICU and several could be adapted or removed. Large adaption required
Impact of Health Information Technology Scale. (I-HIT) [37]	Acceptability	Excellent	Good	CP 6	U- Good [29]	Aimed at acute healthcare staff. OECD developed, no evidence of cost for use	Based on using a specific IT system, however the phrasing of most the questions recommend themselves to use with any specific bundle or intervention. Several questions would require adaption
Evidence Based Practice Questionnaire for Nurses [38]	Acceptability	Fair	Fair	CP 7	U—Good [24]	Developed in the UK, aimed at nurses for use in any healthcare setting. No evidence of cost for use	On initial description, this is focused on the attitude of nurses towards any EBP and its role in routine care, however on reading the scale, this is specifically focused on EBP as a concept and is not adaptable to a specific EBP intervention
Evidence Based Practice Attitude Scale (EBPAS) [33]	Acceptability	N/A	Excellent	CP 5 PAPERS 8	U- Good [15] PAPERS 12	OECD developed, no evidence of cost for use. Based on EBP, not for a specific criterion. Tested on a variety of healthcare staff	Covers four areas of acceptability – openness, appeal, requirements and divergence – based on establishing healthcare workers attitudes towards the acceptance and use of EBP’s. Would require some adaption due to the language used but easy fit for PICU and the ICU liberation bundle
Acceptability of Intervention Measure; Intervention Appropriateness Measure; Feasibility of Intervention (AIM/IAM/FIM) [32]	Acceptability Appropriateness Feasibility	Poor	Poor	CP 8	U- Excellent (4 per measure)	OECD developed. No evidence of cost for use. Not for a specific intervention or setting. Further work on validation in progress so scope for scores to improve	Four questions per measure designed to assess three of the eight taxonomy domains for any intervention that has been implemented. Selected as they are often considered the leading indicators of implementation success. However only tested on people with implementation research experience and the questions may seem similar to healthcare staff with less research experience
Normalisation Measure Development Questionnaire (NoMAD) [39]	Sustainability	Fair	Fair	CP 7	U—Good [23]	OECD developed. Not clear if cost for use. Developed to assess any complex intervention. Tested on a variety of healthcare staff	Based on Normalisation Process Theory (NPT), focusing on implementation embedding and integration. Would work easily with any intervention, including ICU liberation bundle and with a variety of staff involved in the project
Perceived Characteristics of Intervention Scale (PCIS) [40]	Appropriateness Feasibility	N/A	N/A	PAPERS 7	PAPERS 13	Developed in the USA., aimed at evaluating EBP for mental health treatments. Tested on a mix of mental health professionals. No evidence of cost for use	Based on the implementation model initially developed by Everett Rogers, then expanded and elaborated on by Greenhalgh (2004). Covers several concepts including relative advantage, compatibility, complexity and potential for reinvention. Specifically written to be able to be adapted to different EBP and therapies so with minor adaption could work well with interventions such as the ICU Liberation Bundle in the PICU environment

#### Instrument analysis

The included instruments were then read in greater detail, considering all the instruments item by item and considering any relevant supplementary material linked to them. Data was extracted from each of the instruments into a document characteristics table (Table 4).

After this further analysis, one of the seven was not adaptable to a PICU context [38] as it focused on the concept of evidence-based practice (EBP) rather than the implementation of EBP interventions and was therefore excluded.

Of the remaining six, two were formulated for specific populations/interventions [36, 37] but at least half of the questions in each fit the proposed topic and setting well, for example, ‘I feel that I work as part of a team with a recognised and valued contribution’ [36] and both scored highly for methodological and psychometric strength. However, both would require reasonable adaption and there is the potential that the level of adaption would reduce the methodological quality of them. Thus, the remaining instruments were mapped to the CFIR domains to provide insight into the extent each instrument considers different aspects of implementation (Table 5).

**Table 5:** Instruments mapped to the CFIR domains and constructs

Intervention Characteristics	Outer Setting	Inner Setting	Characteristics of Individuals	Intervention Process
**Intervention Source**	**Patient Needs and Resources**	**Structural characteristics**	**Knowledge and beliefs about the intervention** I-HIT [37] EBPAS [33] AIM/IAM/FIM [32] NoMAD [39] PCIS [40]	**Planning** EBPAS [33] PCIS [40]
**Evidence strength and quality** EBPAS [33]	**Cosmopolitanism**	**Network and communications** Person- Centred Healthcare for Older Adults (PCHCOA) [36] I-HIT [37] EBPAS [33] NoMAD [39]	**Self-efficacy** PCHCOA [36] I-HIT [37] AIM/IAM/FIM [32] NoMAD [39] PCIS [40]	**Engaging** NoMAD [39]
**Relative Advantage** I-HIT [37] NoMAD [39] PCIS [40]	**Peer pressure**	**Culture** PCHCOA [36] AIM/IAM/FIM [32] NoMAD [39] PCIS [40]	**Individual stage of change** I-HIT [37] EBPAS [33] NoMAD [39]	**Executing** NoMAD [39] PCIS [40]
**Adaptability** PCHCOA [36] AIM/IAM/FIM [32] NoMAD [39] PCIS [40]	**External policies and incentives** EBPAS [33]	**Implementation Climate** PCHCOA [36] I-HIT [37] EBPAS [33] NoMAD [39] PCIS [40]	**Individual identification with the organisation** PCHCOA [36] AIM/IAM/FIM [32] NoMAD [39]	**Reflecting and Evaluating** NoMAD [39] PCIS [40]
**Trialability** PCIS [40]		**Readiness for implementation** I-HIT [37] EBPAS [33] AIM/IAM/FIM [32] NoMAD [39] PCIS [40]	**Other personal attributes** EBPAS [33]	
**Complexity** I-HIT [37] AIM/IAM/FIM [32] NoMAD [39] PCIS [40]				
**Design quality and packaging** I-HIT [37] EBPAS [33] PCIS [40]				
**Cost**				

### Results from mapping to the CFIR

On analysing the mapped CFIR (Table 5), the majority of the instruments focus predominately on the ‘inner setting’ and ‘characteristics of the individuals’ domains with limited focus outside of these two domains. In particular, the PCHCOA survey [36] and the I-HIT scale [37] have minimal reach outside of these domains. The PCHCOA survey [36] was validated in Australia and has the highest psychometric score [12]. However, it focuses on the provision of good quality care rather than the implementation of an intervention. Whilst this is a beneficial area to measure, it does not fit the required purpose of measuring staff's understanding and attitudes towards a complex intervention. The I-HIT scale [37] was validated in the United States with strong psychometric scores [12] and is aimed at an acute healthcare setting which would be a better fit with PICU, particularly as PICU is a high-technology environment. However, the focus is clearly on a specific type of information technology that may not be found in every healthcare setting and as such would potentially require significant adaption. The level of adaption that both these instruments would require has the potential to reduce their reliability. Furthermore, they are the two longest scales, and neither consider aspects such as the process of the intervention or understanding of the evidence base. As such, they were excluded.

Of the remaining four, the combined scales by Weiner et al. (2016) have the lowest psychometric scores and do not cover either the ‘outer setting’ or ‘process of intervention’ domains. Furthermore, it was only tested by implementation science researchers and psychologists who had implementation research experience in the United States. There is no evidence of testing by staff from any other healthcare profession or who work in acute healthcare settings. On reading the measures, the statements in each (that are measured using the Likert scale) could seem similar to each other if presented to a healthcare professional with no experience in implementation science. For example, in the Feasibility of Intervention Measure, two of the four statements are ‘Intervention seems doable’ and ‘Intervention seems possible’. This could run the risk of respondents not recognising the intended difference between the statements and giving an answer that is not representative of their true opinion of the intervention. The combination of these factors and the low COSMIN score means that these instruments will be excluded from the shortlist.

Of the three remaining scales, only the EBPAS [33] covered all five domains. Whilst neither the PCIS scale [40] nor the NoMAD questionnaire [39] covered the outer setting, they both provided a much more thorough coverage of the ‘process of intervention’ domain, including questions on reflection and evaluation which would be very beneficial for the required purpose as it is recognised that successful adoption of an intervention is more likely if adequate feedback is provided and the benefits can be seen [41]. EBPAS would require some adaption as it was written in the United States utilising more American terminology. This combined with the poorer psychometric scores means that it will be excluded from the shortlist. The NoMAD was validated in the UK using a wide range of healthcare professionals from different healthcare settings and as such would require minimal adaption for use in a PICU setting. Whilst the PCIS was developed in the USA, using mental health professionals, it was specifically designed to be adaptable to any evidence-based intervention and would also require minimal adaption. Both of these instruments have the potential to be further developed and validated for  an acute healthcare setting.

## Discussion

This SR of SRs identified five SRs, all of which utilised clear, rigorous and replicable methods. They also all provided a thorough assessment of the methodological and psychometric strength of the included instruments with a breakdown provided of what had been tested with each instrument. However, the Clinton-McHarg et al. SR [24] was the only review not to provide a score for this, just providing a pass/fail mark instead meaning that there was less clarity about the quality of the testing. The pragmatic scoring undertaken had some weaknesses, with only two reviews [21, 22] scoring every instrument for pragmatic factors such as the language used and cost of the instrument. However, the Allen et al. SR [21] only analysed the 38 measures that were deemed to be fully or partially transferrable to other settings. Whilst analysing and highlighting the transferability of those instruments is useful for those seeking to adapt an instrument for a different setting (and this was the only review to consider this aspect) it could be argued that analysing all the identified instruments would have provided beneficial information.

The reviews were rigorous in so far as, they all led to variations of the same conclusion, despite variations in methodology and healthcare/policy setting. This was because the majority of included instruments showed inadequate or poor evidence of psychometric strength and in order to progress the use of such measures, further psychometric research on them is required. This supports what has been identified in the implementation research literature, that whilst the theory and exploratory research in the field of implementation outcomes is strengthening and expanding, work on measures used in practice is much cruder [4]. The SRs from both Khadjesari et al. [12] and Clinton-McHarg et al. [24] specifically recommend further developing an existing instrument, rather than developing new ‘ad hoc’ instruments. This supports both the literature and the second phase of this SR of SRs.

The use of the CFIR in the second stage was beneficial in enabling further analysis of the instruments and a decision-making process to identify the most appropriate for use within acute healthcare settings, such as PICU. The finding that the majority of the instruments most closely mapped to the ‘inner setting’ and the ‘characteristics of individuals’ was in keeping with the findings from the original Clinton McHarg et al. SR [24]. This suggests that the focus of measures to date aims more to understand the immediate environment where the innovation had been implemented, rather than considering the broader scope of the implementation that would be covered by the lesser-used domains ‘outer setting’ and process of intervention’. This is further supported by the recent scoping review by Proctor et al. [2] which found that most included implementation outcome studies only looked at the implementation of a singular intervention into a specific environment. As such they did not capture the real way in which healthcare organisations function to try and deliver, implement and sustain multiple interventions. It has been suggested that any new measures created should consider all CFIR domains to give greater breadth and depth of understanding of the factors that impact the implementation of evidence into practice [24] and further aid the building of an implementation knowledge base across settings [20]. This thought process can be further expanded to consider that if work is to be done, such as this study, to identify existing outcome measurement instruments for use, then one that covers more of the domains will provide more benefit for use across different interventions and healthcare settings.

The decision-making process, following the use of the CFIR, identified two potential instruments for use, the PCIS scale [40] and the NoMAD questionnaire [39]. Both instruments cover similar areas, considering recognition of the benefit of the intervention, training and resources, so it would not be beneficial to use both as a pair due to the repetition that would incur. However, both have slight areas of difference in the questions asked that would provide the researcher with useful information. For example, NoMAD considers stakeholder support which was highlighted as a key element needed for successful implementation of a complex intervention in the PICU [10] and healthcare settings in general [42] and further recognised as a vital aspect when considering undertaking the measurement of implementation outcomes [3]. This is not covered in PCIS, but PCIS has a stronger section on trialability and adaptability, which has the benefit of identifying whether the intervention can be tested, reversed if necessary and adapted to meet local needs [20]. The importance of this is highlighted in literature identifying both the challenges involved in de-implementing interventions that have proved to have minimal effect [9] and also identifying that an intervention that was implemented successfully in one healthcare setting will not necessarily transfer to a different setting with the same success [43]. As such, it is not immediately clear which of the two instruments is preferable over the other. As a result, the recommendation for further research is to further develop and validate both instruments for use in acute healthcare settings, and undertake work to identify which would be most suitable for use within the PICU setting.

### Strengths and limitations

This SR of SR provides a novel insight into existing work on implementation outcome measurement instruments. The work undertaken to identify an existing tool to be adapted for use with implementing new EBP and interventions into acute healthcare settings, such as the PICU environment, uses a comprehensive and rigorous process throughout both phases, including the data searching, assessment of methodological quality and the creation of the secondary eligibility criteria, which could be utilised by other researchers seeking to find a tool for use in another healthcare setting.

A limitation is that there is a potential that by excluding three SRs when searching for specific implementation outcome measures, some valid instruments may have been overlooked. However, firstly it was noted that there was some crossover of instruments, with some being referenced in more than one SR, suggesting that those with any transferability were being identified by good-quality search criteria. Secondly, by applying strict criteria, it enabled the research team to undertake a more thorough and in-depth look at the selected instruments.

## Conclusion

This SR of SRs used a novel and rigorous two-phased approach to synthesise data from five SRs to establish the methodological strength of implementation outcome measurement instruments and to apply further criteria to identify validated instruments that could be eligible for use in acute healthcare settings. The methodological quality of the implementation outcome measurement instruments was found to be inadequate, highlighting the need to focus on undertaking further validation research on existing instruments so that they can be used for a variety of EBP interventions in healthcare settings, rather than creating new instruments for a single intervention. The use of the CFIR enabled two instruments to be identified that cover multiple domains and have scope to be adapted for use when implementing evidence-based practice in acute healthcare settings such as the PICU. Further research is necessary to help close the literature gap identified in this paper but this SR of SRs provides a strong starting point. Work will be undertaken to select, adapt and further validate an instrument for use in practice in the PICU setting. Furthermore, the methodology used in this review could be adapted by other researchers to identify instruments suitable for use in other healthcare settings.

## Data Availability

The data used to analyse the instruments within the systematic reviews has been made available by the authors of each of the five included systematic review and can be accessed via their online journal publications, see the reference list.
